# Characterization of an Aerosol-Based Photobioreactor for Cultivation of Phototrophic Biofilms

**DOI:** 10.3390/life11101046

**Published:** 2021-10-05

**Authors:** Dorina Strieth, Andreas Weber, Johannes Robert, Judith Stiefelmaier, Jonas Kollmen, Marianne Volkmar, Michael Lakatos, Volkmar Jordan, Kai Muffler, Roland Ulber

**Affiliations:** 1 Bioprocess Engineering, University of Kaiserslautern, 67663 Kaiserslautern, Germany; stiefelmaier@mv.uni-kl.de (J.S.); kollmen@mv.uni-kl.de (J.K.); volkmar@mv.uni-kl.de (M.V.); ulber@mv.uni-kl.de (R.U.); 2 Math2Market, 67655 Kaiserslautern, Germany; andreas.weber@math2market.de; 3 Chemical Engineering, University of Applied Sciences Münster, 48565 Steinfurt, Germany; johannes.robert@fh-muenster.de (J.R.); jordan@fh-muenster.de (V.J.); 4 Integrative Biotechnology, Applied Logistics and Polymer Sciences, University of Applied Sciences Kaiserslautern, 66953 Pirmasens, Germany; michael.lakatos@hs-kl.de; 5 Department of Biotechnology, Enzyme and Fermentation Technology, Bioprocess Technology and Fundamentals of Microbiology, University of Applied Sciences Bingen, 55411 Bingen, Germany; k.muffler@th-bingen.de

**Keywords:** bioengineering, biofilm, cyanobacteria, photosynthesis, antibiotics, neurodegenerative disease, photobioreactor

## Abstract

Phototrophic biofilms, in particular terrestrial cyanobacteria, offer a variety of biotechnologically interesting products such as natural dyes, antibiotics or dietary supplements. However, phototrophic biofilms are difficult to cultivate in submerged bioreactors. A new generation of biofilm photobioreactors imitates the natural habitat resulting in higher productivity. In this work, an aerosol-based photobioreactor is presented that was characterized for the cultivation of phototrophic biofilms. Experiments and simulation of aerosol distribution showed a uniform aerosol supply to biofilms. Compared to previous prototypes, the growth of the terrestrial cyanobacterium *Nostoc* sp. could be almost tripled. Different surfaces for biofilm growth were investigated regarding hydrophobicity, contact angle, light- and temperature distribution. Further, the results were successfully simulated. Finally, the growth of *Nostoc* sp. was investigated on different surfaces and the biofilm thickness was measured noninvasively using optical coherence tomography. It could be shown that the cultivation surface had no influence on biomass production, but did affect biofilm thickness.

## 1. Introduction

Cyanobacteria are ubiquitous, phototrophic prokaryotes. They are often considered as a promising source of new therapeutic molecules, since they are able to synthesize a variety of bioactive compounds from different molecule classes. The described activities of molecules from cyanobacteria include antifungal, antiviral, antibacterial, algicidal, and anti-inflammatory properties. Furthermore, some cyanobacterial phycobiliproteins, which make up to 60% of total cellular protein, are described to have anti-Alzheimer’s disease activity or be neuroprotective. For example, Chaubey et al. reported the therapeutic potential of an isolated phycobiliprotein against neurodegenerative diseases in silico, in vitro and in vivo [1].

In their natural habitat, terrestrial cyanobacteria grow as phototrophic biofilms. They live embedded in a self-produced matrix made of extracellular polymeric substances (EPS), which serve as nutrients [2] and water storage [3,4] and act as protection barriers against biotic and abiotic conditions. The cultivation of terrestrial cyanobacteria in submerged systems is possible, but changes in the cell morphology and the EPS are described in the terrestrial cyanobacterium *Nostoc flagelliforme*, which results in a low biomass concentration (0.335 g L^−1^ [5]) and cell vitality [6]. Cultivation of terrestrial cyanobacteria as a biofilm leads to higher growth rates compared to submersed cultivations [7,8,9]. Furthermore, the cultivation of phototrophic microorganisms like terrestrial cyanobacteria as biofilms can make biotechnological processes easier and more efficient. Various studies have shown that an artificial biofilm can achieve cell densities of up to 300 g L^−1^ [10,11,12,13,14,15,16]. High cell densities in combination with the protective EPS lead to a high level of robustness and resilience to fluctuating and suboptimal conditions [17]. Additionally, no chemicals are required for immobilization, since phototrophic biofilms naturally settle on surfaces [17]. The product purification of secreted substances is facilitated by immobilized cells and higher productivity can be achieved in continuous processes [18], since the growth rate can be decoupled from the dilution rate. 

The requirements for phototrophic biofilm reactors differ in some points from those for submerged reactors. Since the microorganisms grow immobilized on the surface, the choice of a suitable material is important for the adhesion of the biofilm. Therefore, the hydrophobicity of the cultivated microorganisms must be determined and a suitable biocompatible material with appropriate surface properties (hydrophilic or hydrophobic) must be selected in order to facilitate the adhesion of the biofilm. The pH also plays an important role in biofilm formation [19]. For example, cells adhere better in an alkaline than in an acidic medium [20]. Differently from heterotrophic biofilm formers, phototrophic biofilms perform photosynthesis. This means an optimal CO_2_ supply is essential. When CO_2_ is dissolved in water, the pH decreases. Therefore, a regulation of pH is essential for optimal adhesion of the biofilm on substrates. Similar to submersed photobioreactors, the availability of minerals in the medium is directly dependent on the pH. For example, basic cations (Mg^2+^, Ca^2+^) are available at low proton concentrations and mainly cationic acids (iron, cadmium, aluminium) are available at high proton concentrations. The development of biofilm PBRs face major challenges, but the imitation of natural habitats increases productivity of the cells [21,22,23]. To imitate conditions in the desert or the rain forest an aerosol-based PBR was developed by Kuhne et al. [24].

In this work an aerosol-based photobioreactor was characterized for the cultivation of terrestrial cyanobacteria as a biofilm and compared to previous prototypes [7,18]. Based on the experimental data, models for the distribution of light, temperature and aerosol (supplying minerals and water for the biofilm) were developed. Different surfaces were characterized regarding hydrophobicity, contact angle, light and temperature distribution. Additionally, the growth of the terrestrial cyanobacterium *Nostoc* sp. was investigated on various surfaces. Optical coherence tomography (OCT) was used to record noninvasively the biofilm thickness during the cultivation. 

## 2. Materials and Methods

### 2.1. Setup of the Emersed PhotoBioReactors (ePBRs)

Three ePBRs could be operated simultaneously and were placed under high power LEDs (EPSW-VF66 BIN, Edison-opto, New Taipei City, Taiwan) in an incubator (IPS 749, Memmert, Germany) to control the temperature. The aerosol for ePBR 1 was produced by a Pariboy SX (Pary, Starnberg, Germany) and aerosols for ePBR 2 and ePBR 3 were provided by a vaporizer unit using ultrasound.

### 2.2. Determination of the Aerosol RTD in the ePBR

To determine the residence time distribution (RTD) of aerosol particles in the ePBR at a gas volume flow of 1 L min^−1^ a negative step function was used. Therefore, the tracer (aerosol particles) was pumped into the reactor until the entry concentration coincided with the exit concentration (steady state). At the start time, the flow of aerosol was interrupted. The aerosol density was captured in black and white images using a camera (HCC-1000, VDS Vosskühler GmbH, Osnabrück, Germany, three pictures per second, exposure time 10.8 ms). The histograms (grey scale distribution) of the recorded images were read out using ImageJ [25] and then an average was calculated for each image, which was used as a reference for the concentration of the aerosol particles in the reactor. However, the smallest mean grey value stands for a reactor that contains no aerosol. This value was subtracted from all the images taken and the resulting grey value *n* was assumed to be the amount of substances *c*, at time *t*. Subsequently, for the calculation of the aerosol concentration in the reactor, the amount of the substances was divided by the reactor volume *V_R_*, of 0.171 L. However, it is assumed that the standardized tracer concentration (aerosol) can only assume values between one and zero, since c(t) < n_0_ with n_0_ = the mean grey value at the time t_0_ applies all the time. The test time t is replaced by the dimensionless time θ_t_.

### 2.3. Simulation of Aerosol Distribution in the ePBR

CFD (Computational Fluid Dynamics) simulation was used to determine the aerosol distribution via the software GeoDict (developed by Math2Market GmbH, Kaiserslautern, Germany). Simulation parameters are described in the supporting data.

### 2.4. Surface Characterization

To determine the surface roughness of different substrates, an atomic force microscope (AFM, XE-70, Park System, Suwon, South Korea) was used in true noncontact mode. The contact angel was measured optically using an OCA 15 plus (OCA 15plus, Dataphysics, Filderstadt, Germany). BG11-medium was used for the determination of the contact angle, because this medium was used in cultivations.

### 2.5. Temperature and Light Distribution 

The ePBR was placed in an incubator for 24 h at 24 °C. Subsequently, the temperature was measured using a laser thermometer (Laserliner Thermospot, Umarex gmbH, Arnsberg, Germany) and for the light distribution, a quantum sensor (LI-COR LI-190R, LI-COR, Lincoln, Nebraska, USA) was used. Light and temperature were determined at defined distances from the light source. The cold light LEDs were set to 10,000 µmol_photons_ m^−2^ s^−1^ in all reactor tests. The light distribution was simulated by the ray tracing method of Robert et al. [26]. Calculations for the temperature distribution are available as supporting data. 

### 2.6. Preculture

*Nostoc sp.* is a terrestrial cyanobacterium from the desert soil in Nizzana (Israel) and was provided by Prof. Dr. Burkhard Büdel (Department of Plant Ecology and Systematics, University of Kaiserslautern, Germany). Precultures were cultivated for 10 days to reach the exponential phase in a shaking incubator (Multitron S-000115689, Infors HT, Bottmingen, Switzerland) at 120 rpm with 2.5 eccentricities at 30 °C and continuous lightning at 100 µmol_photons_ m^−2^ s^−L^ in 300 mL shaking flasks without chicanery, containing 50 ml of standard BG11 medium.

### 2.7. Cultivation in the ePBR

The preculture was centrifuged for 15 min at 8000× *g* (centrifuge 383K, Hermle Labortechnik GmbH, Wehingen, Germany). The supernatant was discarded, and the biomass pellet was used to inoculate the ePBR with a 60 mg cell wet weight (CWW) per square centimeter. Light intensities varied with the used surface between 9–63 (borosilicate glass); 19–85 (PMMA) and 7–31 (silicone) µmol_photons_ m^−2^ s^−1^, with a day/night rhythm of 14/10 h and a constant temperature at 24 °C. BG11 medium was continuously supplied as an aerosol. A correlation between CWW and cell dry weight (CDW) was done using the biomass from preculture to convert the CWW used for inoculation of the ePBR into CDW. After two weeks of cultivation, biomass was scraped off the surfaces and used for EPS extraction. CWW was lyophilized for 24 h at −20 °C and 1 mbar and CDW was determined gravimetrically. Biomass productivity is given as area time yield (ATY) in gram CDW per square meter per day. 

### 2.8. EPS Extraction

EPS were extracted using the physical extraction method with a combination of heat and ultrasonication [27]. Therefore, biomass was resuspended in a 5 mL preheated (60 °C) 0.05 % NaCl solution and incubated in a 50 mL centrifuge tube (Eppendorf 5702 Centrifuge accessories, Sigma-Aldrich) in an overhead shaker for 30 min at 60 °C. Afterwards, the suspension was incubated in an ultrasonic bath (Sonorex Digiplus, Bandelin, Germany) with an intensity of 100 W for 10 min at 20 °C. The suspension was centrifuged for 15 min at 8000× *g* and the supernatant containing the EPS was transferred to a 50 mL tube. The biomass and supernatants were lyophilized for 24 h at −20 °C and 1 mbar. The weight of EPS and CDW were determined gravimetrically. CDW and EPS were added together because EPS belongs to CDW. 

### 2.9. Determination of the Biofilm Thickness Using OCT

**Figure 1:**
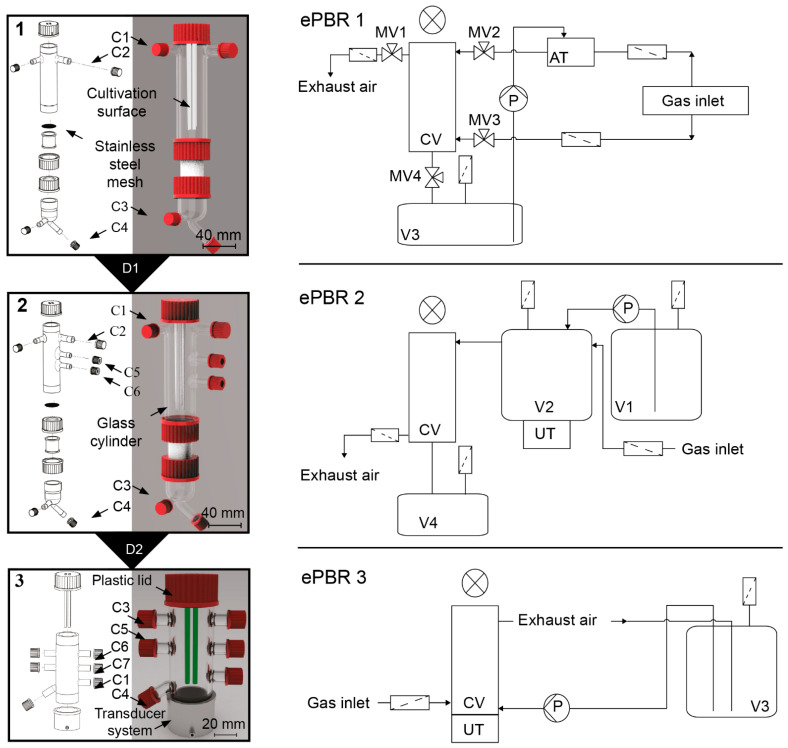
Development steps (D) of the reactor geometry including process flow charts of reactor periphery. (1) ePBR 1 developed by Kuhne et al. [7]. C1 = Inlet for compressed air. C2 = Inlet of aerosol. C3 = Outlet of compressed air and aerosol. C4 = Outlet for recycling medium. (2) Optimized ePBR 2 by Strieth et al. [18]. Two connections (C5 and C6) were added for online monitoring (temperature and humidity). (3) New design with an integrated atomizer unit. Addition of two connections (C7 = Inlet for medium; C4 = Outlet for recycling medium). Process flow charts of the different development steps of the ePBR 1 [7], ePBR 2 [18] and ePBR 3. V1 = Stock vessel for medium. V2 = Nebulizer vessel. V3 = Stock vessel for medium/condensed medium. V4 = Vessel to recycle the condensed medium. UT = Ultrasonic transducer. P = Dosing pump, to pump the medium. CV = Cultivation vessel.

Biofilm thickness was determined using a spectral domain OCT (sdOCT, Thorlabs, Newton, New Jersey, USA). OCT allows noninvasive imaging of the biofilm [28]. To perform the measurements at reproducible points, the ePBR was removed under sterile conditions and placed under the sdOCT. Subsequently, three 2D scans with a 1.5 cm length were recorded on one rod, and two rods per reactor (ePBR 3, see Figure 1) were investigated, resulting in six replicates per reactor. The images were analyzed using ImageJ (Rasband, W.S., ImageJ, U. S. National Institutes of Health, Bethesda, Maryland, USA). Hereby, the cross-sectional area and length of the biofilm on each 2D scan were determined and the average biofilm thickness was calculated.

## 3. Results and Discussion

### 3.1. Development of the ePBR

Up to date, three different ePBRs have been developed. The first prototype of an ePBR for the cultivation of terrestrial cyanobacteria (ePBR 1, see Figure 1) was developed by Kuhne et al. [7] and consists of a glass cylinder in which roughened rods are inserted. The rods are fixed (glued) into the cover and act as an optical fiber and cultivation surface with a cultivation area of 25.12 cm^2^. The medium is given as an unsterile aerosol produced by a Pariboy SX and can be added above the upper connection (C2). Medium is recycled above connection 3 and pumped into the stock vessel (see Figure 1 ePBR 1). Connection 1 and 3 can be either used for gas exchange measurements or for cultivation with different gas compositions. Strieth et al. replaced the unsterile aerosol production system by a vaporizer unit using ultrasound [18,29] and added two connections for the insertion of temperature and humidity sensors (C5 and C6; see Figure 1 ePBR 2). One vaporizer vessel produced the aerosol for three ePBRs (see Figure 1) which led to an irregular aerosol distribution and equal aerosol supply was not guaranteed. For this reason, the geometry of the ePBR 1 developed by Kuhne et al. [7] and optimized by Strieth et al. [18] was changed. Therefore, the ultrasonic transducer was integrated into the reactor. To ensure an optimal supply of the biofilm with nutrients, compressed air distributes the aerosol through connection 4 (see Figure 1 ePBR 3). For a constant quality of aerosol supply, two connections were added to the ePBR 3: (i) C7 = Inlet for medium; (ii) C4 = outlet for medium recycling (see Figure 1). Thereby, a constant water column above the ultrasonic transducer is guaranteed. This results in a simplified reactor periphery (see Figure 1). Three different surfaces were tested as a cultivation surface: (i) sandblasted borosilicate glass, (ii) roughened methyl methacrylate (PMMA) using abrasive paper (grain size: 100 after CAMI (Coated Abrasive Manufacturing Institute) and corresponding to 140 µm) and (iii) untreated silicone. The rods for ePBR 3 are made to fit exactly and can be inserted into the lid according to the modular principle. The silicone hose was installed in the ePBR via a borosilicate glass rod. In the following, the recent prototype, the ePBR 3, was characterized.

### 3.2. Aerosol Distribution in eBPR 3

**Figure 2:**
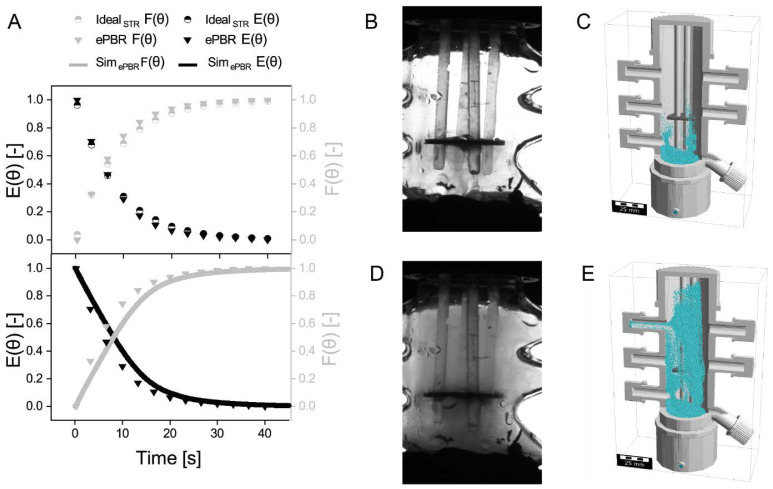
CFD-simulation of aerosol flow through ePBR 3 using an ultrasonic transducer. (**A**) Experimentally determined RTD and CDF for ePBR 3 (ePBR), calculated RTD and CDF for an ideal stirred reactor (Ideal_STR_) using the volume of ePBR 3 and simulated RTD and CDF for ePBR 3 (Sim_ePBR_). E(θ) = dimensionless residence time distribution function. F(θ) = dimensionless cumulative distribution function. For modelling and visualization, the software GeoDict was used. (**B**) Aerosol distribution without compression captured in a black and white image using a camera. (**C**) Simulated aerosol distribution without any volume flow. The particles (cyan) are distributed in the lower third of the ePBR without compressed air. For this reason, the aerosol particles were injected with a slight upward starting movement. (**D**) Aerosol distribution with 1 L min^−1^ compressed air after 30 seconds of running time captured in a black and white image using a camera. (**E**) Simulated aerosol distribution with 1 L min^−1^ compressed air after 30 seconds of running time.

The distribution and residence time of aerosol particles in the ePBR is important since the aerosol supplies the cyanobacteria with nutrients and water. Therefore, a CFD simulation was performed to determine the aerosol distribution. Additionally, the aerosol density was captured experimentally in black and white images using a camera to validate simulated data and to calculate the residence time distribution (RTD). The RTD function E(t) and cumulative distribution function (CDF) F(t) were calculated to compare the simulated data with the experimentally determined data (see Figure 2A). Based on the snapshots of aerosol distribution taken with the camera, a complete mixing of the aerosol in the ePBR was assumed (see Figure 2D). To classify the ePBR between the two limit cases: (i) the ideal flow tube and (ii) the ideal stirred tank reactor, the CDF and RTD functions were calculated based on the reactor dimensions for ideal stirred tank reactors. The course of experimentally determined CDF and RTD functions are similar to the calculated CDF and RTD for ideal stirred tank reactors (see Figure 2A). The trapezoidal rule was used to calculate the mean residence time of aerosol particles and was 5.38 s in the experiment, 6.1 s in the simulation and 6.3 s for an ideal stirred reactor. The difference in the mean residence time of particles can be explained by dead zones in the reactor that were neglected in the calculation for ideal stirred tank reactors. Therefore, the effectively used reactor volume was determined and results in a dead volume of 47.73%. The dead volume was also confirmed visually on the recorded images and was then calculated with ImageJ. For this purpose, the grey values at every position of an image (ePBR with and without aerosol) were read out and then subtracted from each other. The areas outside the reactor were then cut out and not taken into account for the calculation. If the difference in grey values was between 0 and 50, the area was assumed to be dead volume. Finally, an effective reactor volume of 68% could be determined, which is higher than the calculated one. However, by calculating the mean residence time in the ePBR using the effective used volume in the ePBR 3, the difference to an ideal stirred reactor can be decreased (5.8 s). The courses of the experimentally determined and the simulated RTD and CDF functions were nearly identical in their course (see Figure 2). Differences between experimental and simulated RTDs for the aerosol could be caused by assuming slightly different geometries and it should be noted that the images taken in the experiment only allow the concentration to be calculated in two-dimensional space. Due to mutual shading and different depths caused by the cylindrical basic shape of the reactor, the two-dimensional concentration cannot be transferred directly to the volume. Therefore, the two-dimensional concentration can only be regarded as a qualitative indicator of the aerosol particle concentration and may be based in comparison to simulated results. It was not possible to focus on different levels to visualize the aerosol concentration at different points in the reactor. Moreover, the simulation neglected condensation or evaporation of aerosol particles as well as interactions between particles. Simulated aerosol particles touching a surface were assumed to be absorbed into the liquid film. In the experiment, a liquid film on all surfaces could also be noticed, but a precise mechanism of aerosol particle interaction on this film could not be clarified. In addition, a steady flow was simulated in the CFD simulation, which means that the flow was not influenced by the aerosol particles and a stationary vortex formation (which does not move) is imaged. The pronounced vortex formation in the area of cultivation indicates that the biofilm is well supplied with aerosol. In addition, the simulation showed, like the aerosol density captured in black and white with a camera, dead spaces in the connections and in the upper area of the reactor. The same dead spaces were also described by Kuhne in the ePBR 1 [7]. Since those dead spaces have no negative impact on biofilm growth, there is no need for further optimization.

### 3.3. Temperature and Light Distribution in ePBR 3

**Figure 3:**
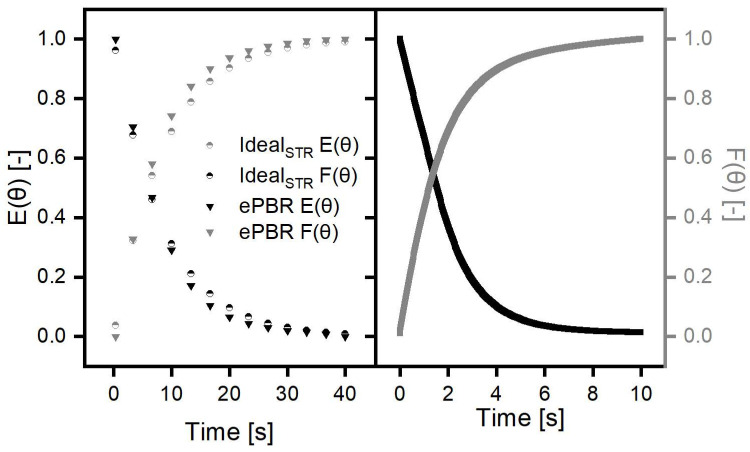
Measured and simulated light and temperature distribution on different substrates. The light intensity was determined using a quantum sensor at defined distances from the light source (6, 8, 10 cm) in the cultivation area. Light intensity of the light source was 10,000 µmol_photons_ m^−2^ s^−1^ and corresponded to the settings at which the cultivation experiments were carried out. Temperature was measured using a laser thermometer at defined distances from the light source (0, 3, 4, 5, 6, 7, 8 cm).

Surface temperature plays a key role in the cultivation of phototrophic organisms, since fluctuation of temperatures can result in dehydration of the biofilm or reduced productivities. For this reason, the temperature distribution on borosilicate glass, PMMA and silicone was investigated and simulated (see Figure 3). The temperatures of the surfaces in the cultivation area range were between 21.8–24.8 °C (borosilicate glass), 20.8–21.8 °C (PMMA) and 21–21.4 °C (silicone) and are within the temperature range tolerated by different cyanobacterial strains [21]. The simulated temperature profiles of the individual substrates show slight deviations compared to those determined experimentally. The deviations for borosilicate glass are ± 0.74 °C, for PMMA ± 1.26 °C and for silicone ± 0.97 °C. The simulation can thus be assumed to be successful and transferred to other surfaces. The highest temperatures in the cultivation area (5–8 cm distance from the light source) were detected on borosilicate glass, which means that the temperature set in the incubation cabinet does not correspond to the temperature on the cultivation surfaces. The lowest temperatures in the cultivation area were found in silicone, which is due to the fact, that the specific heat capacity is lower than that of PMMA and the silicone hose was installed in the ePBR via a borosilicate glass rod. Between silicone and borosilicate glass, the space has a specific heat capacity of 1.005 kJ kg^−1^ K^−1^ which minimizes the heat transfer to the silicone. This is because borosilicate glass has a lower specific heat capacity (0.83 kJ kg^−1^ K^−1^) than PMMA (1.50 kJ kg^−1^ K^−1^) and silicone (1.48 kJ kg^−1^ K^−1^).

In addition to temperature, the availability of light plays a major role for phototrophic organisms. The cultivation surfaces simultaneously serve as optical fibers in the ePBR and thus guarantee the light supply for the organisms. The light distribution depends on the surface roughness, whereby a more constant distribution is achieved in roughened, tapered glass rods [8]. Basically, an increase in the roughness affects the angle of incidence of the light rays at the interface and a stronger refraction towards the outside can be achieved [30]. In this work, the most ideal light distribution was expected on borosilicate glass (R_a_ = 1.168 ± 0.363) followed by PMMA (R_a_ = 0.328 ± 0.087) and silicone (R_a_ = 0.252 ± 0.008) because the surface roughness on borosilicate glass was highest. In addition, the silicone tube was integrated into the reactor via a borosilicate glass rod, which means that the light has to pass through at least two interfaces. Nevertheless, the most uneven light distribution was measured on borosilicate glass with 0.6% of the incident light at a distance of 5 cm from the light source (see Figure 3). This is probably due to the fact, that the first 2 cm of the rod were not roughened and therefore a larger part of the incident light broke out of the rod, which resulted in a more uneven light distribution. For this reason, the amount of light emitted (3–9 cm) was summed up in an Ulbricht sphere. In addition, the percentage loss of light refracted from the rod within the first 3 cm was calculated and was 67.55% for borosilicate glass (= 6507 µmol_photons_ m^−2^ s^−1^) and 65.18 % (= 6259 µmol_photons_ m^−2^ s^−1^) for PMMA. These losses are significantly higher than those which were observed by Robert et al. (20% loss due to mismatch between the incident angle and the opening angle of the rod; 20% loss due to Fresnel reflection at the rod’s surface). This discrepancy is expected due to the smaller rod and the larger LED in comparison to Robert et al. It is expected that a reflective cladding with a low refractive index will improve the light transmission within this section [26]. The highest light intensity was measured on PMMA at a distance of 5 cm from the light source with 0.8% of the incident light. Finally, the light transmission was modelled using a model for ideal matt surfaces and tapered light guides [26]. Although there was a significant difference in the surface roughness of the three rods (borosilicate glass (R_a_ = 1.168 ± 0.363), PMMA (R_a_ = 0.328 ± 0.087), silicone (R_a_ = 0.252 ± 0.079), all the measured light intensities (black data points) match the simulated data with a high level of precision (see Figure 3). In the rough section of the rod, the light decoupling patterns are similar to cylindrical TiO_2_ coated rods [31,32]. It is concluded that surfaces with a roughness of (0.3 < R_a_ < 0.9) can be considered as ideally matt and scattering. Furthermore, this model can be applied not only for simple scattering surfaces, but also for more complex surfaces, as the silicone/PMMA composed surfaces are [33]. However, the inner surface needs to be rough enough to scatter the light rays. The emitted light intensities of the silicone covered PMMA rod are considerably lower in comparison with the uncovered PMMA rod, because some photons are reflected at the inner surface of the silicone tube and some photons are absorbed in the silicone layer according to the Beer–Lambert law. It is concluded that the silicone covered rods are not desirable for future reactor designs, since there is no broadening of the light distribution, but a considerable loss of photons. The tapering of the rods is very useful. Whereas almost 20 % of the photons were lost at the axial end using cylindrical rods of the same length [26], there was almost no residual photon flux at the axial end using tapered rods. 

Based on the results, the conditions for phototrophic biofilms on PMMA should be the best, since the highest light intensities in the cultivation could be measured here. However, it should be noted that the glass cylinder of the reactor throws scattered light back onto the surface and ambient light enters the reactor (about 30 µmol_photons_ m^−2^ s^−1^). In summary it can be said that the light intensities in the cultivation area (5–8 cm distance from the light source) are lower than the light compensation point of *Nostoc* sp. (formerly listed incorrectly as *Trichocoleus sociatus*) 90 ± 30 µmol_photons_ m^−2^ s^−1 [21]^) without taking scattered light into account. However, if the scattered light of 30 µmol_photons_ m^−2^ s^−1^ is added to the transmitted light intensities, the light intensities are in the cultivation range for the substrates PMMA (49–115 µmol_photons_ m^−2^ s^−1^) and borosilicate glass (39–93 µmol_photons_ m^−2^ s^−1^) and in the area of the light compensation point. Only for the silicone surfaces (37–61 µmol_photons_ m^−2^ s^−1^) are the light intensities below the light compensation point (see Figure 3).

### 3.4. Comparison of the Prototypes

To investigate the influence of different development steps of ePBRs the growth of *Nostoc* sp. was examined under equal conditions. The growth is given as ATY in gram CDW per square meter per day. With these optimizations, the ATY could be increased from 0.83 ± 0.39 g_CDW_ m^−2^ d^−1^ (ePBR A) to 2.4 ± 0.36 g_CDW_ m^−2^ d^−1^ (ePBR C).

### 3.5. Influence of Different Substrates on Biofilm Formation

The substrate plays a crucial role in the primary adhesion of the biofilm. It is known that the adhesion of cyanobacteria to solid surfaces is primarily dependent on the properties of the EPS [34]. However, there is no general statement about the relationship between cell adhesion and surface hydrophobicity. Some organisms have adapted to adhesion to hydrophobic and others to hydrophilic surfaces [35]. In this work, two hydrophilic (borosilicate glass and PMMA) and one hydrophobic surface (silicone) (see Table 1) were examined with regard to the ATY, content of EPS and pigment composition. The hydrophobicity of the surface depends inter alia on the surface roughness. High roughness encourages the adhesion of biofilms due to the enlargement of the surface and reduces, for example, shear forces occurring in submerged systems such as flow cells [36]. Schlegel showed an increase in the space time yield (STY) proportional to the surface roughness of heterotrophic biofilms [37]. This was also expected for *Nostoc* sp. since the cells had hydrophilic properties after surface-associated and submerged cultivation (data not shown). Due to the surface properties of the substrates used and the hydrophilic polysaccharides of the EPS, the highest ATY was expected on borosilicate glass, followed by PMMA and silicone. However, it should be noted here that the cultivation surfaces simultaneously act as optical fibers and thus the thermal conductivity and light distribution also have an impact on the productivity of the biofilm. For example, borosilicate glass has the highest thermal conductivity and therefore the highest temperatures in the cultivation area (5–8 cm from the light source), followed by silicone and PMMA. Although silicone has a lower thermal conductivity than PMMA, it was installed in the reactor via a borosilicate glass rod, which explains the higher temperatures (see Table 1). The light distribution depends on the material and the surface quality and was best for the cultivation surface PMMA, followed by borosilicate glass and silicone. The light compensation point of *Nostoc* sp. is 90 ± 65 µmol_photons_ m^−2^ s^−1^ [21]. If stray light is included, the substrates consisting of PMMA and borosilicate glass cover this area.

**Figure 4:**
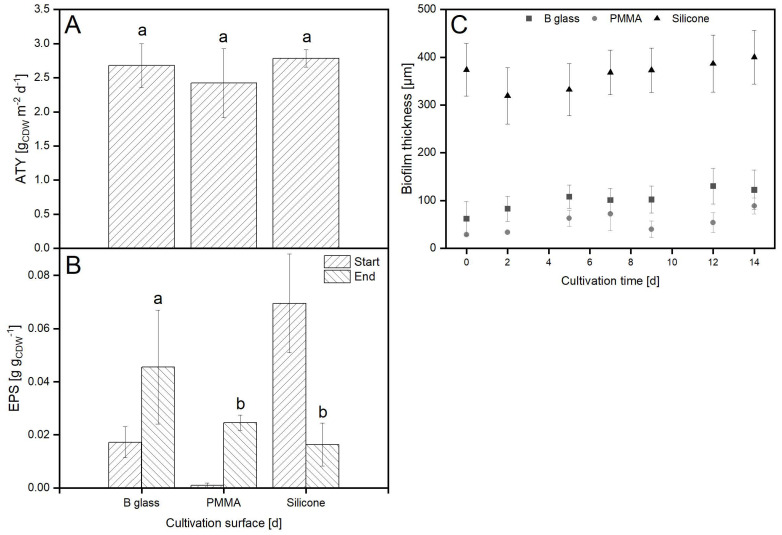
Influence of different substrates (**A**) on biofilm growth, (**B**) EPS production and (**C**) biofilm thickness. The Area Time Yield ATY (A), and the start and end values of the contents of EPS (B) are shown. Test parameters: Cultivation time = 14 d, Light intensity = 9–63 (B glass); 19-85 (PMMA); 7-31 (silicone) µmol _photon_s m^−2^ s^−1^; Day/Night Rhythm = 14/10 h; Temperature = 24 ° C, BG11 medium; 24 hours aerosol supply. The thickness was determined using OCT (n = 10). Statistics: Mann–Whitney-U test (two-sided, 5%). Significant differences are visualized using different letters.

Based on the characteristics of the surfaces and the hydrophilic properties of *Nostoc* sp., PMMA should be the best substrate of the examined surfaces for the cultivation of *Nostoc* sp. However, no differences in the ATY achieved could be found on the different substrates (see Figure 4A). Especially on silicone, a lower ATY was expected compared to the other surfaces, due to the low light intensity in the cultivation area and the hydrophobic surface. Presumably the way of inoculation plays an important role as well. The CWW was centrifuged out of the submerged preculture and then applied to the surfaces. Sekar showed that the amount of adherent cells from *Nitzschia amphibia* (diatoms) is proportional to the cell density, regardless of the wettability of the surface [20]. Preliminary experiments showed that *Nostoc* sp. actively changes the pH of the medium, which means that the precultures have a pH of nine (data not shown), which also promotes cell adhesion. Accordingly, the conditions for the adhesion of *Nostoc* sp. on the different surfaces were almost optimal. Furthermore, it can be assumed that the composition of the EPS changed over the cultivation period to improve the adhesion. The synthesis of rhamnolipids from EPS could, for example, increase the surface hydrophobicity of the cells [38] and thereby lead to an improvement in cell adhesion. *Pseudomonas aeruginosa* produces rhamnolipids in order to increase the surface hydrophobicity and thereby adhere better to hydrophobic surfaces [39,40]. Basically, an increase in EPS production on silicone would be expected in order to increase the surface hydrophobicity of the biofilm. However, the EPS amount decreased on silicone and increased on borosilicate glass and PMMA over the cultivation period (see Figure 4B). Hydrophilic polysaccharides may have been degraded to increase the percentage of hydrophobic polysaccharides. For this purpose, the polysaccharides of the EPS should be analyzed in further experiments. The production of EPS on the hydrophilic surfaces could be explained by the fact that the growth conditions were better, whereby nutrients were stored in the EPS [41]. During the inoculation process the equal distribution of biomass on silicone was difficult. This resulted in a higher biofilm thickness at the beginning of the cultivation compared to the hydrophilic surfaces (see Figure 4C). This was probably due to the hydrophobic surface of the silicone and the hydrophilic EPS from *Nostoc* sp. The vertical spread of the biofilm on silicone could have been influenced by two factors: (i) the hydrophobic surface of silicone and (ii) pH of the nutrient mist. The pH in the ePBR, without a suitable buffer or a pH control, drops to pH 6 due to atmospheric CO_2_ dissolving in the water droplets. As previously described, an acidic pH makes the adhesion of the biofilm difficult [20]. The drop in pH in combination with the hydrophobic surface could have caused the vertical spread of the biofilm. It has now been argued that the pH of the culture at pH 9 facilitates the adhesion of the biofilm. It should be noted here that the aerosol is only added after the biofilm has adhered and therefore has no influence on the primary adhesion, but on the horizontal spreading of the biofilm over the cultivation period. In contrast, an area spread on the hydrophilic surfaces was possible, which led to a vertical and horizontal spread of the biofilm (Figure 4C). This could also explain the high production of EPS on hydrophilic surfaces, which facilitated the adhesion of the biofilm. Potentially, less EPS was produced on borosilicate glass compared to PMMA, because of the better wettability of borosilicate glass.

## 4. Conclusions

An aerosol-based photobioreactor was optimized and characterized to cultivate phototrophic biofilms. Different substrates were investigated experimentally regarding hydrophobicity, contact angle, light- and temperature distribution. Further, the results were successfully simulated. The residence time of the aerosol was similar to optimal stirred tank reactors and the simulation showed vortex formation in the area of biofilm cultivation, resulting in an optimal supply to the biofilm. The growth of *Nostoc* sp. was investigated on different surfaces in three ePBR prototypes. Due to the optimizations the growth almost tripled. It could be shown that the cultivation surface had no influence on biomass production but did affect biofilm thickness.

## Figures and Tables

**Table 1:** Summarized characteristics of the substrates used as a cultivation surface. The thermal conductivity, temperature distribution and light intensity are shown in the cultivation area (5–8 cm distance from the light source with 10,000 µmol_photons_ m^−2^ s^−1^).

	Borosilicate Glass	PMMA	Silicon
Thermal conductivity λ [W m^−2^ K^−1^]	1.2	0.19	0.1-0.3
Temperature distributionT_5–8_ [°C]	21.8–24.8	20.8–21.8	21–21.4
Light intensity LI_5–8_ [µmol_photons_ m^−2^ s^−1^]	9–63	19–85	7–31
Square roughness R_q_ [µm]	1.168 ± 0.363	0.328 ± 0.087	0.252 ± 0.079
Contact angle Θ_Y_ [°]	69.3 ± 0.8	79.6 ± 1.7	113.2 ± 0.7

## References

[1] Chaubey M.G., Patel S., Rastogi R.P., Srivastava P.L., Singh A.K., Madamwar D., Singh N.K. (2019). Therapeutic potential of cyanobacterial pigment protein phycoerythrin: In silico and in vitro study of BACE1 interaction and in vivo Aβ reduction. Int. J. Biol. Macromol..

[2] Schooling S.R., Beveridge T.J. (2006). Membrane Vesicles: An Overlooked Component of the Matrices of Biofilms. J. Bacteriol..

[3] Schmitt J., Flemming H.-C. (1999). Water binding in biofilms. Water Sci. Technol..

[4] Hobley L., Ostrowski A., Rao F.V., Bromley K.M., Porter M., Prescott A., MacPhee C., van Aalten D., Stanley-Wall N.R. (2013). BslA is a self-assembling bacterial hydrophobin that coats the Bacillus subtilis biofilm. Proc. Natl. Acad. Sci. USA.

[5] Yu H., Jia S., Dai Y. (2008). Growth characteristics of the cyanobacterium *Nostoc* flagelliforme in photoautotrophic, mixotrophic and heterotrophic cultivation. Environ. Boil. Fishes.

[6] Gao K., Ye C. (2003). Culture of the terrestrial cyanobacterium, Nostoc flagelliforme (Cyanophyceae), under aquatic conditions1. J. Phycol..

[7] Kuhne S., Strieth D., Lakatos M., Muffler K., Ulber R. (2014). A new photobioreactor concept enabling the production of desiccation induced biotechnological products using terrestrial cyanobacteria. J. Biotechnol..

[8] Kuhne S. (2015). Fermentation von Phototrophen Organismen zur Produktion von Biotechnologischen Wertstoffen.

[9] Gustavs L., Schumann R., Eggert A., Karsten U. (2009). In vivo growth fluorometry: Accuracy and limits of microalgal growth rate measurements in ecophysiological investigations. Aquat. Microb. Ecol..

[10] Nowack E.C.M., Podola B., Melkonian M. (2005). The 96-Well Twin-Layer System: A Novel Approach in the Cultivation of Microalgae. Protist.

[11] Johnson M.B., Wen Z. (2009). Development of an attached microalgal growth system for biofuel production. Appl. Microbiol. Biotechnol..

[12] Ozkan A., Kinney K., Katz L., Berberoglu H. (2012). Reduction of water and energy requirement of algae cultivation using an algae biofilm photobioreactor. Bioresour. Technol..

[13] Christenson L.B., Sims R.C. (2012). Rotating algal biofilm reactor and spool harvester for wastewater treatment with biofuels by-products. Biotechnol. Bioeng..

[14] Blanken W., Janssen M., Cuaresma M., Libor Z., Bhaiji T., Wijffels R.H. (2014). Biofilm growth ofChlorella sorokinianain a rotating biological contactor based photobioreactor. Biotechnol. Bioeng..

[15] Liu T., Wang J., Hu Q., Cheng P., Ji B., Liu J., Chen Y., Zhang W., Chen X., Chen L. (2013). Attached cultivation technology of microalgae for efficient biomass feedstock production. Bioresour. Technol..

[16] Boelee N.C., Janssen M., Temmink H., Taparavičiūtė L., Khiewwijit R., Jánoska A., Buisman C.J.N., Wijffels R.H. (2013). The effect of harvesting on biomass production and nutrient removal in phototrophic biofilm reactors for effluent polishing. Environ. Boil. Fishes.

[17] Muffler K., Lakatos M., Schlegel C., Strieth D., Kuhne S., Ulber R. (2014). Application of Biofilm Bioreactors in White Biotechnology. Productive Biofilms.

[18] Strieth D., Schwing J., Kuhne S., Lakatos M., Muffler K., Ulber R. (2017). A semi-continuous process based on an ePBR for the production of EPS using Trichocoleus sociatus. J. Biotechnol..

[19] Liehr S.K., Eheart J.W., Suidan M.T. (1988). A modeling study of the effect of pH on carbon limited algal biofilms. Water Res..

[20] Sekar R., Venugopalan V.P., Satpathy K.K., Nair K.V.K., Rao V.N.R. (2004). Laboratory studies on adhesion of microalgae to hard substrates. Hydrobiologia.

[21] Lakatos M., Strieth D., Cánovas F.M., Lüttge U., Matyssek R. (2018). Terrestrial Microalgae: Novel Concepts for Biotechnology and Applications. Progress in Botany Volume 79.

[22] Strieth D., Ulber R., Muffler K. (2017). Application of phototrophic biofilms: From fundamentals to processes. Bioprocess Biosyst. Eng..

[23] Podola B., Li T., Melkonian M. (2016). Porous Substrate Bioreactors: A Paradigm Shift in Microalgal Biotechnology?. Trends Biotechnol..

[24] Kuhne S., Strieth D., Weber A., Muffler K., Lakatos M., Ulber R. (2013). Screening of two terrestrial cyanobacteria for biotechnological production processes in shaking flasks, bubble columns, and stirred tank reactors. Environ. Boil. Fishes.

[25] Schneider C.A., Rasband W.S., Eliceiri K.W. (2012). NIH Image to ImageJ: 25 years of image analysis. Nat. Methods.

[26] Robert J., Jüstel T., Ulber R., Jordan V. (2020). Modelling and Experimental Investigation of Luminous Coupling in UVLED Driven Optical Fiber Reactors. J. Photocatal..

[27] Strieth D., Stiefelmaier J., Wrabl B., Schwing J., Schmeckebier A., Di Nonno S., Muffler K., Ulber R. (2020). A new strategy for a combined isolation of EPS and pigments from cyanobacteria. Environ. Boil. Fishes.

[28] Stiefelmaier J., Strieth D., Di Nonno S., Erdmann N., Muffler K., Ulber R. (2020). Characterization of terrestrial phototrophic biofilms of cyanobacterial species. Algal Res..

[29] Schmidt T., Just L. (2009). Device and Method for the Cultivation and Production of Biological Material in a Nutrient Mist. U.S. Patent.

[30] Ehrbar P. (2000). Rechnergestützter Entwurf von Beleuchtungssystemen mit Starren Lichtleitern. Ph.D. Dissertation.

[31] Wang W., Ku Y. (2002). The light transmission and distribution in an optical fiber coated with TiO2 particles. Chemosphere.

[32] Peill N.J., Hoffmann M.R. (1998). Mathematical Model of a Photocatalytic Fiber-Optic Cable Reactor for Heterogeneous Photocatalysis. Environ. Sci. Technol..

[33] Xu J., Ao Y., Fu D., Lin J., Lin Y., Shen X., Yuan C., Yin Z. (2008). Photocatalytic activity on TiO2-coated side-glowing optical fiber reactor under solar light. J. Photochem. Photobiol. A Chem..

[34] Neu T.R., Marshall K.C. (1990). Bacterial Polymers: Physicochemical Aspects of Their Interactions at Interfaces. J. Biomater. Appl..

[35] Pringle J.H., Fletcher M. (1983). Influence of substratum wettability on attachment of freshwater bacteria to solid surfaces. Appl. Environ. Microbiol..

[36] Characklis W.G., McFeters G.A., Marshall K.C. (1990). Physiological Ecology in Biofilm Systems.

[37] Schlegel C. (2016). Produktive Biofilme auf mikrostrukturierten Metalloberflächen.

[38] Zhang Y., Miller R.M. (1994). Effect of a Pseudomonas rhamnolipid biosurfactant on cell hydrophobicity and biodegradation of octadecane. Appl. Environ. Microbiol..

[39] Yuan X., Ren F., Zeng G., Zhong H., Fu H., Liu J., Xu X. (2007). Adsorption of surfactants on a Pseudomonas aeruginosa strain and the effect on cell surface lypohydrophilic property. Appl. Microbiol. Biotechnol..

[40] Zhong H., Zeng G.M., Yuan X.Z., Fu H.Y., Huang G.H., Ren F.Y. (2007). Adsorption of dirhamnolipid on four microorganisms and the effect on cell surface hydrophobicity. Appl. Microbiol. Biotechnol..

[41] Mager D.M. (2010). Carbohydrates in cyanobacterial soil crusts as a source of carbon in the southwest Kalahari, Botswana. Soil Biol. Biochem..

